# Bridging Ancestry-Stratified Bias in Pharmacogenomics AI: Toward Metabolomics-Inclusive Multi-Omics Precision Medicine

**DOI:** 10.3390/jpm16060332

**Published:** 2026-06-20

**Authors:** Heayyean Lee, Khadijah Sajid, Dayeon Lee

**Affiliations:** 1 Plamica Labs, Batten Hall, 125 Western Ave, Allston, MA 02163, USA; dayeon.lee@plamica.com; 2 Department of Internal Medicine, St Luke’s Health Network, 421 Chew St., Allentown, PA 18102, USA

**Keywords:** pharmacogenomics, algorithmic bias, metabolomics, multi-omics, precision medicine, drug response prediction, health equity, cross-population transferability, federated learning, pharmacometabolomics

## Abstract

Pharmacogenomics AI offers significant potential for individualized drug therapy; however, its clinical benefits remain unevenly distributed. Models trained predominantly on European-ancestry data consistently underperform in non-European populations, with polygenic risk scores (PRS) showing an estimated 39–73% reduction in predictive accuracy in African-ancestry cohorts across complex traits. These disparities have driven increased interest in moving beyond single-layer genomic approaches. Multi-omics frameworks integrating genomic, transcriptomic, proteomic, and metabolomic data have emerged as a promising strategy to improve prediction across heterogeneous clinical populations, as each molecular layer provides distinct and complementary biological information. Among these layers, metabolomics may represent a particularly transferable component across populations. Metabolite profiles capture the downstream functional output of biological systems influenced by genetic, environmental, dietary, and microbiome-related factors, and may therefore be less reliant on ancestry-stratified allele frequency structures that underlie performance disparities in genomic models. This review synthesizes evidence regarding the mechanistic basis of genomic bias in pharmacogenomics AI, the emerging role of multi-omics integration, especially metabolomics, in improving predictive performance, and the current landscape of computational strategies for bias mitigation, including federated learning, transfer learning, domain adaptation, and synthetic data generation. Collectively, current evidence supports metabolomics-inclusive multi-omics frameworks as a biologically plausible, hypothesis-generating strategy to reduce reliance on ancestry-linked genomic features. However, direct evidence that such frameworks reduce ancestry-related bias in clinical AI outputs remains limited, underscoring the need for globally diverse datasets and prospective multi-population validation.

## 1. Introduction

The convergence of artificial intelligence (AI) and precision medicine is transforming drug therapy through scalable analysis of high-dimensional molecular data, with pharmacogenomics serving as a critical molecular foundation for predicting drug efficacy, toxicity, and individualized dosing. Early advances were driven by large-scale pharmacological screening resources such as the Genomics of Drug Sensitivity in Cancer (GDSC) and the Cancer Cell Line Encyclopedia (CCLE), which established foundational benchmarks for machine learning-based genotype-phenotype prediction [1,2,3]. Since then, a range of advances, including population-scale resources such as the UK Biobank [4], ancestrally diverse initiatives such as the NIH All of Us Research Program [5], and genomic foundation models pre-trained across millions of DNA sequences [6], have markedly expanded the scale, representativeness, and predictive capacity of available genomic training resources. These cumulative advances are accelerating the integration of AI-driven pharmacogenomic tools into increasingly diverse clinical settings [7].

**Figure 1:**
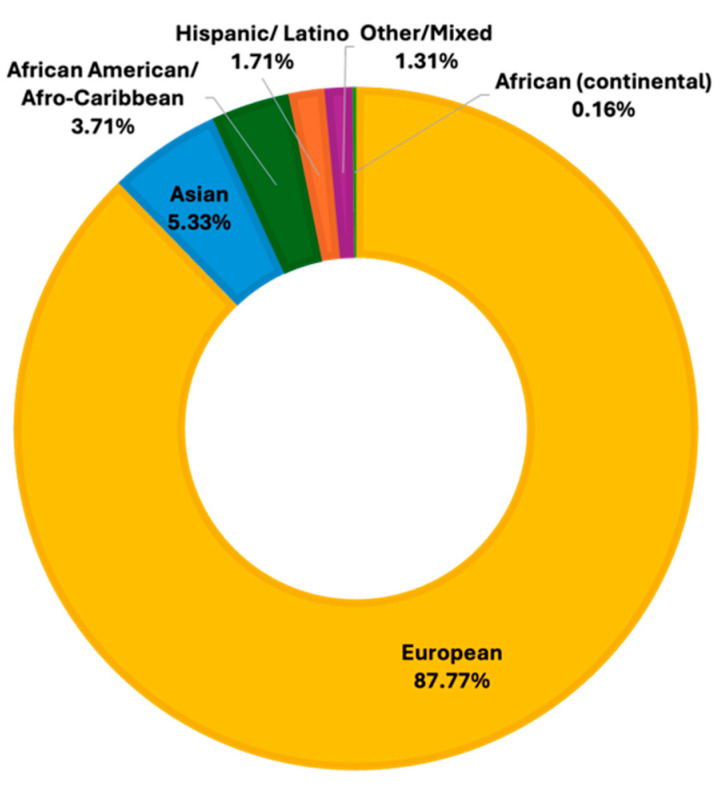
Distribution of GWAS participants by ancestry (2025). Data derived from the GWAS Diversity Monitor (https://www.gwasdiversitymonitor.com/; accessed on 11 May 2026).

Yet a fundamental equity problem pervades this field: the genomic datasets that train these AI models are overwhelmingly derived from individuals of European ancestry. As of mid-2025, European participants account for approximately 87–88% of all GWAS participants, while all other ancestry groups remain markedly underrepresented, with continental African populations below 1% [8,9]. This representation imbalance remains substantial in contemporary GWAS datasets (Figure 1). As a result, pharmacogenomic AI models often generalize poorly across ancestry groups; European-derived polygenic risk scores lose approximately 39–73% of predictive accuracy in African-ancestry cohorts, exemplifying clinically consequential algorithmic bias [10].

Multi-omics integration, the combined analysis of genomic, transcriptomic, proteomic, and metabolomic data layers, has increasingly been explored as a strategy to mitigate limitations of single-omics pharmacogenomics. Integrating multiple molecular layers may more effectively capture aspects of drug-response biology that are not fully represented by any single modality alone [11]. Each layer contributes distinct yet complementary molecular information: germline genomics provides relatively stable variant–drug response associations; transcriptomics captures dynamic regulatory states and disease-specific expression patterns; proteomics reflects functional protein abundance and post-translational modifications relevant to drug target engagement; and metabolomics profiles the downstream biochemical output of these upstream processes within the patient’s physiological context. Metabolomics, in particular, may offer a functionally broader molecular layer across heterogeneous clinical settings, as it reflects the downstream functional state of biological systems formed by both genetic and environmental influences. Unlike ancestry-stratified germline variants, it captures both genetic and non-genetic influences, including environmental, microbiome, and lifestyle factors. When incorporated into multi-omics AI frameworks, it may help reduce reliance on ancestry-specific genomic features, although further population-level validation remains needed.

This review examines ancestry-related bias in AI-driven pharmacogenomics and evaluates the emerging rationale for metabolomics-inclusive multi-omics integration as a strategy for improving cross-population generalizability. Although the primary focus is pharmacogenomics and pharmacometabolomics, selected examples from adjacent precision-medicine settings are included where they help illuminate whether metabolomics-informed prediction remains reproducible across heterogeneous cohorts, sites, or population groups. At the current stage of the literature, these examples are intended to be hypothesis-generating rather than definitive evidence that metabolomics-inclusive models reduce ancestry-related bias in clinical AI outputs. Relevant literature was identified through iterative searches of PubMed, Scopus, and Google Scholar, supplemented by citation tracking of relevant articles, using combinations of terms related to pharmacogenomics, pharmacometabolomics, metabolomics, multi-omics integration, artificial intelligence, machine learning, bias, fairness, ancestry, race, ethnicity, cross-population generalizability, and precision medicine. Studies were selected based on relevance to ancestry-related bias, cross-population or cross-site validation, metabolomics-informed prediction, and clinical or translational significance. Formal risk-of-bias assessment, study weighting, and quantitative meta-analysis were not performed.

## 2. Sources of Bias in AI-Driven Pharmacogenomics

### 2.1. Eurocentric Genomic Datasets and the Representation Gap

The foundational source of bias in pharmacogenomics AI is the persistent underrepresentation of non-European populations in major genomic resources, which systematically skews the evidence base used to train and validate predictive models. Similar patterns extend to pharmacogenomic databases and clinical trial cohorts, further limiting model generalizability across populations. At the level of individual pharmacogenes, this representation gap leads to clinical inaccuracies in predictions. African-ancestry populations exhibit the highest CYP2D6 haplotype diversity, with multiple distinct haplotype blocks, whereas European populations have a single predominant block [12]. This uncharacterized diversity translates directly into phenotypic uncertainty: among Sub-Saharan African individuals, 35% are classified as having an indeterminate CYP2D6 metabolizer status, compared with <0.09% in other global populations.

Large-scale sequencing data from the All of Us Research Program further reinforce this pattern, identifying substantial pharmacogenomic variation across populations, particularly in admixed American and African-ancestry groups [5]. Population-level studies also demonstrate substantial inter-ethnic variability in CYP2C19 metabolizer status, with non-normal metabolizer frequencies ranging from 32% in Mexico to 80% in India [13].

### 2.2. Population Structure and Dataset Shift

Beyond simple underrepresentation, cross-population performance gaps in pharmacogenomic AI are also driven by population structure and dataset shift. Differences in allele frequencies, linkage disequilibrium architecture, environmental exposures, and clinical covariates mean that associations learned in one ancestry group may not transfer reliably to another. Consequently, models trained predominantly in European cohorts may capture ancestry-specific statistical proxies rather than more biologically stable predictive signals. These limitations suggest that fairness in pharmacogenomic AI requires more than simply increasing sample diversity. Recent approaches such as PhyloFrame indicate that explicitly modeling ancestry-related structure, rather than treating it solely as a confounder, can improve prediction performance across populations [10]. Such findings highlight the need for methods that jointly address representation imbalance and population-specific genomic frameworks. For example, genotype-guided warfarin dosing algorithms developed largely in European populations performed substantially worse in African American patients because clinically relevant variants such as CYP2C9*5, *6, *8, and *11 were not included in the original models [14,15]. This case shows how dataset shift can translate directly into clinical dosing errors.

## 3. Multi-Omics Integration for Drug Response Prediction

The promise of AI-driven pharmacogenomics rests on the quality and completeness of molecular information fed into predictive models. In the current research landscape, that information is primarily genomic and transcriptomic in origin, and models optimized on ancestry-stratified germline data may not generalize well to populations whose allele-frequency architectures differ from the training distribution [16].

### 3.1. Genomics and Transcriptomics as the Current Predictive Backbone

Genomics and transcriptomics have formed the central backbone of pharmacogenomics AI due to their strong empirical foundation and widespread data availability. Decades of GWAS have established robust associations between germline variants and inter-individual variability in drug metabolism, efficacy, and toxicity, with support from large-scale biobank resources such as the UK Biobank, in which 99.5% of individuals carry variants predicted to influence drug response [17,18]. Transcriptomics further contributes a dynamic regulatory layer, capturing tissue-specific and disease-contextual gene expression states relevant to drug sensitivity. This combined genomic–transcriptomic framework has demonstrated strong predictive performance. Integrative models such as MOLI and DeepCDR have shown improved drug-response prediction compared to single-omics approaches, while more advanced architectures, including graph neural network-based models, have further extended these gains [19,20,21].

Despite their strong predictive value, genomics and transcriptomics alone may not fully capture the multidimensional determinants of drug response required for equitable precision medicine. Germline variants in drug-metabolizing enzymes, transporters, and drug targets account for important but incomplete portions of interindividual variability, whereas transcriptomic states provide dynamic regulatory information without fully capturing downstream biochemical physiology. Drug response is also determined by diet, microbiome composition, environmental exposures, concomitant medications, and disease-associated metabolic reprogramming factors that may not be directly inferred from genomic or transcriptomic data alone [22].

Moreover, allele frequencies, linkage disequilibrium patterns, and haplotype structures remain strongly stratified across ancestry groups, increasing the risk that models developed on imbalanced datasets learn population-specific genomic proxies rather than shared biological determinants of drug response [16]. These considerations underscore the need to expand multi-omics frameworks to include additional functional layers that may improve robustness and equity across diverse populations.

### 3.2. Metabolomics as a Dynamic Functional Layer for Cross-Population Translation

**Figure 2:**
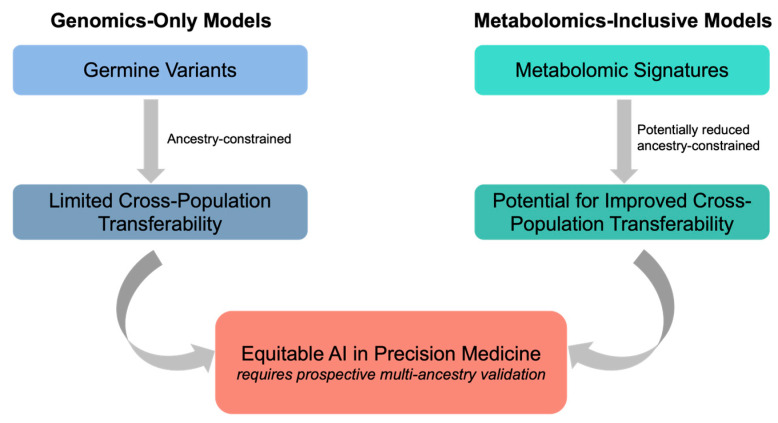
Conceptual framework illustrating the potential role of metabolomics-inclusive AI in cross-population precision medicine.

Metabolomics occupies a conceptually distinct position among omics layers because it measures the realized biochemical output of the organism rather than its genetic potential. It reflects enzyme activity, metabolic flux, pathway utilization, and downstream product concentrations, integrating the combined effects of genetic variation, environmental exposures, diet, microbiome metabolism, and co-medication interactions. In pharmacogenomics, this functional orientation is mechanistically significant: metabolomic profiling of CYP-substrate metabolites can quantify enzyme activity independently of genotype, capturing non-genetic influences that genomic ADME models cannot represent [23]. Unlike ancestry-stratified germline variants, metabolomic profiles are dynamic and environmentally modifiable, providing a complementary source of pharmacological information. From a population perspective, this property may enhance portability across heterogeneous cohorts, as metabolomic signatures are not fixed by ancestry in the same way that germline allele frequencies are. When integrated into multi-omics AI frameworks, metabolomics may reduce reliance on ancestry-specific genomic features [11]. Emerging studies in oncology suggest that metabolomic features can improve drug response prediction and provide clinically relevant biological insights when combined with other omics layers. Conceptually, metabolomics-inclusive frameworks may support improved cross-population transferability by incorporating functional biochemical features that may be less ancestry-constrained than germline variants (Figure 2).

### 3.3. AI Architectures for Integrative Multi-Omics Modeling

Integrating heterogeneous omics layers within AI frameworks while preserving modality-specific information and capturing biologically meaningful cross-omics interactions remains a key challenge in multi-omics modeling. To address this, three major integration strategies have been developed: early (feature-level), late (decision-level), and intermediate (embedding-level) [11]. Early integration concatenates omics features prior to model training and is straightforward to implement, but it remains vulnerable to the curse of dimensionality, along with the loss of modality-specific structure. Late integration trains separate models for each omics modality and subsequently combines their outputs, providing greater flexibility but potentially limiting the capture of biologically meaningful cross-omics interactions. Late and intermediate integration strategies, represented by models such as MOLI, DeepCDR, and MOMLIN, learn modality-specific or compressed latent representations prior to integration, thereby improving management of high-dimensional data while preserving complex nonlinear relationships across modalities [19,21,24]. Recent transformer-based and graph-based multimodal architectures have further expanded the ability of intermediate integration frameworks to model higher-order biological dependencies and cross-omics interactions [20,25]. Across benchmark comparisons, these architectures have consistently outperformed single-omics and conventional early-integration approaches in drug-response prediction tasks. Table 1 summarizes representative multi-omics AI architectures for drug response prediction, including integrated omics layers, model design, validation datasets, and reported performance metrics.

## 4. Algorithmic Bias Auditing Framework

Even with improved biological representation through multi-omics integration, equitable clinical deployment cannot be assumed without explicit algorithmic auditing. Multi-omics models may still inherit bias from imbalanced training cohorts, site-specific practice patterns, or population differences in data quality and missingness, making subgroup-level evaluation essential [29].

Model performance should therefore be assessed separately across ancestry groups, sex, age strata, and clinically relevant subpopulations rather than relying solely on aggregate metrics. In pharmacogenomics, even modest prediction errors may contribute to unsafe dosing or adverse drug events, particularly for therapies with narrow therapeutic windows such as warfarin [30,31]. Recent benchmarking studies further show that genomic prediction models developed predominantly in European-ancestry cohorts often experience measurable performance decline when transferred to African, South Asian, or admixed populations [16].

In addition to discrimination, calibration across populations is also critical because models may systematically overestimate or underestimate risk in specific patient groups. Multicenter evaluations have demonstrated that models with strong internal performance may still exhibit calibration drift after external deployment, emphasizing the importance of recalibration and ongoing monitoring [32]. At the same time, emerging multi-omics foundation models suggest that more stable cross-cohort generalization may be achievable when external validation is incorporated. For example, the SeNMo model maintained nearly identical survival prediction performance between internal and independent external oncology cohorts (C-index 0.760 vs. 0.758), supporting the feasibility of more transferable multi-omics systems [33].

For high-stakes clinical decision-making, predictive systems should communicate not only classification outputs but also calibrated uncertainty, particularly for rare or underrepresented molecular profiles. Interpretability methods such as SHAP values and pathway-level feature attribution may help clinicians assess whether predictions are supported by biologically plausible signals, including CYP variants, inflammatory transcriptomic states, or metabolomic indicators of impaired drug clearance [34,35]. Recent multimodal biomedical AI studies have explored whether uncertainty-aware architectures may improve robustness to missing or heterogeneous omics modalities by dynamically weighting modality reliability during inference [36]. Representative considerations for auditing equitable multi-omics pharmacogenomics AI systems are summarized in Table 2.

## 5. Computational Strategies for Bias Mitigation in Multi-Omics AI

Several computational strategies may help reduce performance disparities in AI-driven precision medicine while more representative global multi-omics datasets are being developed. Rather than replacing the need for diverse data collection, these approaches aim to improve cross-population generalizability, preserve privacy, and mitigate structural imbalance in current training resources [37,38]. Transfer learning and domain adaptation are among the most direct approaches for improving model portability across populations. Models pretrained on large majority-population cohorts can be fine-tuned using smaller underrepresented datasets, allowing broadly shared biological patterns to be retained while adapting to population-specific variation. Domain adaptation methods further aim to align feature distributions between source and target populations, thereby reducing errors caused by ancestry-related dataset shift [37]. These approaches may be especially relevant for multi-omics precision medicine, where sequencing platforms, metabolomic assays, and clinical workflows frequently differ across institutions [39]. Recent population-aware frameworks such as PhyloFrame illustrate that explicitly modeling genetic structure can improve predictive equity across ancestry groups [10].

Federated learning offers a complementary strategy by enabling collaborative model training across hospitals, biobanks, and countries without transferring raw patient-level data. This is particularly relevant in settings where privacy regulations, cost-intensive assays, and fragmented sample collections often limit the assembly of centralized datasets [38]. By allowing geographically distributed institutions to contribute to shared model development, federated frameworks may accelerate the construction of more globally representative multi-omics AI systems. However, successful deployment requires methods that can accommodate heterogeneous data quality, non-identically distributed populations, and unequal cohort sizes across participating sites [37,40].

Generative and synthetic data approaches may also help address underrepresentation by augmenting scarce minority-population datasets with statistically realistic molecular profiles. In principle, conditional generative models may help model ancestry-associated genomic or metabolomic variation, thereby supporting training in low-resource settings [41]. However, synthetic augmentation should be approached with caution: statistical realism does not guarantee biological validity or clinical fairness, and poorly validated synthetic data may amplify rather than reduce existing bias [42]. Overall, these computational strategies are best understood as interim accelerators, not substitutes for prospective recruitment of ancestrally diverse cohorts, standardized multi-omics data generation, and direct real-world validation of equitable precision medicine systems [29,41].

## 6. Translational Clinical Evidence for Multi-Omics AI Across Diverse Populations

Because equitable precision medicine ultimately depends on whether biologically informative features remain reproducible beyond discovery cohorts, externally validated translational studies provide an important framework for evaluating the transferability of metabolomics-inclusive AI across heterogeneous populations.

### 6.1. Oncology

Recent oncology studies increasingly suggest that metabolomics-integrated AI may improve treatment-response prediction by capturing dynamic functional states that extend beyond genomic and transcriptomic information. NSCLC provides one of the most clinically developed examples. Sun et al. integrated plasma metabolomics with machine learning to predict pemetrexed response and identified the tryptophan–kynurenine pathway as the dominant discriminator between responders and resistant patients, achieving an AUC of 0.954 for treatment response prediction [43]. This pathway is biologically plausible and may represent a potentially transferable immunometabolic signal, although cross-ancestry validation has not yet been performed.

Similar findings are emerging in immunotherapy settings. Lee et al. identified amino acid, glycolytic, and bile acid pathways associated with immune checkpoint inhibitor response in NSCLC using targeted metabolomics and machine learning [44]. Metabolomics-based models in lung squamous cell carcinoma have also shown potential for prognostic stratification among patients receiving chemoimmunotherapy, with Zheng et al. developing an eight-metabolite machine-learning model to predict survival in advanced lung squamous cell carcinoma [45]. However, most current studies remain derived from single-center East Asian cohorts, and prospective cross-ancestry validation remains limited.

The strongest direct evidence for cross-racial performance parity in metabolomics AI oncology comes from estrogen receptor-positive (ER+) breast cancer. Santaliz-Casiano et al. demonstrated near-equivalent predictive performance across African American and Non-Hispanic White cohorts (AUCs of 0.79 and 0.78, respectively) despite the models relying on distinct metabolic signatures in each racial group [46]. Rather than indicating race-specific failure, these findings suggest that metabolomics-based AI may adapt to biologically distinct yet clinically relevant metabolic states while maintaining comparable predictive accuracy across racial groups. This adaptive behavior contrasts with the reduced transferability often observed in genomics-only prediction systems and represents one of the clearest current examples supporting the potential equity advantages of metabolomics-inclusive AI.

Cross-site generalizability, another prerequisite for generalizable deployment, has also been demonstrated in ovarian cancer. Ban et al. reported consistently high predictive performance across multiple geographically distinct North American study sites with minimal site-to-site variation, supporting the potential portability of metabolomics-based AI across heterogeneous clinical settings [47]. Additional representative studies published since 2022 are summarized in Table 3.

### 6.2. Metabolic Diseases

Metabolic diseases provide a clinically important setting for evaluating whether metabolomics-integrated AI can improve cross-population prediction beyond genomics alone. Type 2 diabetes (T2D), for example, disproportionately affects racial and ethnic minority populations, while genomics-based prediction models often show limited transferability across ancestries. In contrast, plasma metabolites are strongly shaped by modifiable environmental and lifestyle factors, supporting the potential for more transferable prediction across populations.

Sevilla-González et al. reported that metabolomic variation was predominantly associated with biological and lifestyle factors rather than fixed ancestry in multiethnic cohorts [50]. Importantly, environmentally influenced metabolite and protein mediators were estimated to account for approximately 10–25% of observed racial and ethnic disparities in T2D risk, supporting the potential biological portability of metabolomics-informed prediction across populations. This observation supports the concept that metabolomic profiles may reflect downstream functional physiology shaped by both inherited and modifiable influences, rather than ancestry-stratified genomic architecture alone.

Population-specific discovery studies further demonstrate that metabolomics can preserve both biological specificity and cross-population relevance. Reynolds et al. identified ancestry-enriched metabolite associations in Hispanic/Latino populations, highlighting that admixed metabolomes contain layered biological signals that may be incompletely captured by uniform reference models [51]. Similarly, Chen et al. identified 307 metabolites associated with incident T2D among African American participants in the Jackson Heart Study, with 144 associations replicating in the multiethnic MESA cohort [52]. The inclusion of metabolomic features improved prediction performance from a c-statistic of 0.74 to 0.81, supporting the value of discovery in underrepresented populations, combined with external multiethnic validation.

Direct evidence for cross-ancestry transferability has also emerged in diabetic complications. He et al. demonstrated that metabolomics-based machine learning models for diabetic kidney disease maintained improved predictive performance during external validation from a multiethnic Southeast Asian cohort to the predominantly European UK Biobank cohort (AUC 0.838 vs. 0.743 internally; AUC 0.791 vs. 0.691 in external validation) [53]. Together, these findings suggest that metabolomics-inclusive AI may support more biologically transferable and population-adaptive prediction in metabolic disease settings. Selected recent studies published since 2022 are summarized in Table 4.

### 6.3. Infectious Disease/Immunology

Infectious and immune-mediated diseases provide a particularly strong framework for evaluating metabolomics-integrated AI because host-response metabolic profiles capture dynamic immunometabolic states beyond fixed ancestry labels. COVID-19 represents one of the most extensively characterized examples. Meta-analyses spanning 22 cohorts identified reproducible perturbations in amino acid, bile acid, and TCA cycle pathways, with metabolic dysregulation scaling consistently with disease severity [59]. In a separate targeted plasma multi-omics study, an integrated model using 10 proteins and five metabolites predicted COVID-19 patient survival at hospital admission with 92% accuracy and an ROC-AUC of 0.97 [60]. Additional studies showed that metabolite-based classifiers remained robust across different pandemic waves and treatment contexts, supporting the transferability of core host-response metabolic signatures beyond specific viral variants or demographic groups [61].

Tuberculosis provides one of the strongest examples of cross-population validation in infectious disease metabolomics. Collins et al. identified a plasma kynurenine/tryptophan plus retinol signature that achieved AUCs of 0.93–0.97 across independent cohorts in Ethiopia, South Africa, and Georgia, while maintaining performance regardless of HIV co-infection status. Longitudinal decreases in signature scores during treatment further supported its utility as a dynamic monitoring biomarker [62]. Complementary multi-cohort studies additionally identified lipid biomarkers with reproducible diagnostic performance across external datasets, suggesting that metabolomics may capture conserved host immunometabolic responses across heterogeneous populations and clinical contexts [63].

Studies from tropical and resource-limited settings provide additional evidence supporting cross-population deployment. In melioidosis, metabolomics-based models validated in rural Southeast Asia achieved AUCs of 0.87 for diagnosis and 0.91 for mortality prediction, with kynurenine pathway activation again emerging as a dominant signal [64]. Similar convergence of kynurenine-associated metabolic dysregulation has been reported across sepsis, COVID-19, tuberculosis, and melioidosis [62,64,65], supporting the possibility of a conserved immunometabolic inflammatory axis across multiple severe infectious diseases. Ensemble machine learning studies in sepsis further identified kynurenine, bile acids, and related metabolites as predictors of 28-day mortality, supporting the role of metabolomics for biologically informed risk stratification in critical illness [65].

Emerging evidence from immune-mediated diseases further supports the portability of metabolomics-based prediction across heterogeneous clinical settings. In rheumatoid arthritis, Tang et al. developed a six-metabolite machine-learning classifier that maintained robust diagnostic performance across five independent cohorts and multiple analytical platforms, including seronegative disease subgroups [66]. Together, these studies suggest that metabolomics-inclusive AI may capture conserved, functionally relevant host-response biology with potential for more transferable and equitable prediction across infectious and immune-mediated diseases. Selected recent studies published since 2022 are summarized in Table 5.

### 6.4. Broader Clinical Applications of Metabolomics-Inclusive AI

Additional clinical domains further support the concept that metabolomics-integrated AI may overcome limitations of proxy-based and ancestry-dependent prediction frameworks by capturing the functional biochemical state of the individual. Although not a direct pharmacogenomic application, cardiovascular disease provides one of the clearest recent examples. In a racially and geographically diverse cohort including Black American, White American, and Chinese participants, Deng et al. demonstrated that a metabolite risk score was significantly associated with incident coronary heart disease and improved risk discrimination beyond conventional cardiovascular risk factors [68]. Importantly, predictive performance remained broadly consistent across race, sex, socioeconomic status, and lifestyle strata, supporting cross-population reproducibility of metabolomics-based cardiovascular risk prediction.

Nephrology provides a particularly relevant example of bias introduced by proxy-based clinical measures. Creatinine-based eGFR calculations are influenced by demographic and physiologic factors, including muscle mass and diet, contributing to long-standing disparities in kidney disease risk assessment. Interpretable metabolomics-based machine learning models have demonstrated strong performance for identifying renal dysfunction and metabolite features associated with glomerular filtration rate across internal and external validation cohorts [69]. Unlike creatinine-based demographic corrections, metabolomic profiling may provide a more direct functional assessment of renal physiology, supporting the development of less ancestry-dependent prediction systems.

Pharmacometabolomics further highlights limitations of genotype-centered prediction approaches. In psychiatry, Pardiñas et al. demonstrated substantial ancestry-associated variability in clozapine metabolism across five biogeographical ancestry groups, while polygenic scores explained only a limited proportion of pharmacokinetic variation [70]. These findings support the rationale for metabolomics-based CYP phenotyping, which directly captures functional metabolism beyond ancestry reference norms. Similarly, microbiome-metabolomic machine learning models for SSRI response demonstrated promising predictive performance in internal testing cohorts, supporting treatment stratification approaches based on dynamic host-response biology in addition to genotype-based prediction frameworks [71].

Maternal-fetal medicine provides an additional setting in which metabolomics-based prediction may improve early risk stratification across independent validation cohorts. Metabolite-based machine learning models have demonstrated early prediction of preeclampsia before overt clinical onset, with validation AUCs ranging from 0.753 to 0.885 across cohorts [72,73]. Together, these studies suggest that metabolomics-inclusive AI may support more transferable and physiologically grounded prediction across diverse precision medicine domains. While not direct pharmacogenomic applications, these findings provide additional support for incorporating metabolomics as a complementary functional layer within multi-omics precision medicine frameworks. Selected recent studies published since 2022 are summarized in Table 6.

## 7. Conclusions and Future Directions

The evidence synthesized in this review supports a reconceptualization of the equity challenge in AI-driven precision medicine. The problem is not solely one of representation in genomic discovery cohorts, but also one of feature choice: models built predominantly on ancestry-linked germline variants inherit the limitations of those variants, including reduced transferability across populations and limited ability to capture dynamic determinants of health. Across oncology, metabolic disease, infectious disease, nephrology, cardiovascular medicine, and pharmacometabolomics, recurrent host-response signals, including kynurenine pathway activation, TCA-cycle perturbation, and lipid remodeling, suggest that metabolomic profiles may capture biologic information that could be less constrained by genetic ancestry than germline variant features alone [59,62,68]. This review proposes that incorporating such signatures as a complementary functional layer alongside genomic data may support the development of AI models with improved transferability in precision medicine. It should be noted, however, that reduced ancestry constraint in metabolomic features represents a necessary biological precondition for—rather than direct evidence of—reduced ancestry-related bias in AI model outputs; the latter requires prospective head-to-head demonstration of equitable predictive performance that remains largely absent from the current literature.

Several translational implications emerge from this framework. Because metabolomic profiles reflect the integrated effects of genetics, diet, microbiome ecology, inflammation, medication exposure, and environmental context [77], they may help improve prediction in populations for whom ancestry-matched genomic reference resources remain limited. Pharmacometabolomic phenotyping further offers a functional approach to treatment individualization by directly capturing the metabolic consequences of drug action and CYP activity in the individual patient rather than relying exclusively on ancestry-dependent pharmacogenomic reference models. Beyond treatment individualization, these metabolomics-informed approaches may also help identify biologically relevant pathways and candidate biomarkers that could inform future drug-development efforts. More broadly, the ability of metabolomics to identify biologically distinct subgroups within shared clinical diagnoses may expand precision medicine into areas where genotype-based stratification alone has shown limited clinical utility. Importantly, these approaches are best viewed as complementary to genomics rather than replacements for existing multi-omics frameworks.

Several limitations should also be acknowledged. The conclusions drawn here are based on heterogeneous studies that differed substantially in cohort composition, analytical platforms, validation rigor, and study design, and direct head-to-head comparisons between genomics-only and metabolomics-inclusive fairness metrics, the standard required to demonstrate bias reduction rather than merely improved feature transferability, remain limited. Moreover, many metabolomics-inclusive AI systems remain at the discovery or early validation stage, and prospective evidence for equitable clinical implementation across diverse populations is still emerging. Future progress will require prospective multi-ancestry validation studies, harmonized metabolomics acquisition and preprocessing pipelines, and clinically deployable frameworks that support portable multi-omics prediction across heterogeneous healthcare settings. Ultimately, whether metabolomics-inclusive AI can meaningfully improve cross-population generalizability and support more equitable precision medicine will depend on rigorous real-world validation across the full diversity of patient populations these systems are intended to serve.

## Figures and Tables

**Table 1:** Representative AI architectures for multi-omics drug response prediction in cancer pharmacogenomics.

Model	Omics	Model Type	Cohort	Metric	Ref.
MOLI	Mutation, CNV, expression	DNN (late integration + triplet loss)	GDSC	Improved external validation	[19]
GraphCDR	Mutation, expression, CNV + Drug structure	GNN + Contrastive learning	GDSC	Improved vs. prior methods	[20]
DeepCDR	Mutation, expression, Methylation + Drug graph	Hybrid GCN	GDSC, CCLE	R^2^ ≈ 0.90	[21]
MOMLIN	Clinical, Mutation, expression, TME, Pathways	Sparse correlation + Integration	Breast cancer cohort	AUC = 0.989 (+10% vs. MOFA)	[24]
Pathformer	Multi-omics + Pathway prior + Liquid biopsy	Pathway-informed transformer	TCGA, plasma	+6.3–14.7% F1	[25]
AGMI	Whole-genome omics	GNN (GeNet) + Attention	CCLE, GDSC	+8.3–34.2% vs. SOTA	[26]
MOICVAE	Genomics + Transcriptomics + CNV	Collective variational autoencoder	GDSC, CCLE, TCGA	AUC = 0.856–0.910	[27]
Metabolomics integrated DNN	Expression, CNV, Mutation, RPPA, Metabolomics	DNN + Graph attention	CCLE, GDSC	R^2^ = 0.90	[28]

Abbreviations: DNN, deep neural network; GCN, graph convolutional network; GNN, graph neural network; CNV, copy number variation; TME, tumor microenvironment; RPPA, reverse phase protein array; MOFA, multi-omics factor analysis; SOTA, state-of-the-art; CDR, cancer drug response; AUC, area under the ROC curve.

**Table 2:** Representative Auditing Considerations for Multi-Omics Pharmacogenomics AI.

Domain	Assessment Focus	Example Metrics	Clinical Relevance
Subgroup Performance	Performance across ancestry, sex, and age groups	AUC, MAE, F1	Detects hidden disparities
Calibration	Agreement between predicted and observed risk	Calibration slope, Brier score	Prevents systematic overdosing
Uncertainty	Reliability in rare or out-of-distribution patients	Entropy, conformal interval	Supports informed clinical decision-making in rare presentations
Interpretability	Biological plausibility of model drivers	SHAP, pathway attribution	Supports clinician trust
External Validation	Generalizability across hospitals or populations	Transport AUC	Evaluates real-world robustness
Temporal Drift	Performance stability over time	Recalibration frequency	Detects protocol or population shifts

Abbreviations: AUC, area under the receiver operating characteristic curve; MAE, mean absolute error; SHAP, SHapley Additive explanations.

**Table 3:** Selected 2022–2026 oncology studies supporting metabolomics-inclusive AI for equitable precision medicine.

Indication	Cohort/Design	Key Findings	Cross-Population Relevance	Ref.
NSCLC chemotherapy	323 NSCLC subjects (discovery *n* = 122; validation *n *= 201) Targeted plasma metabolomics Random forest prediction of pemetrexed response	Kynurenine pathway metabolites (KTR, XKR) strongly associated with pemetrexed efficacy Response prediction AUC 0.954	Single-center East Asian cohort Tryptophan–kynurenine pathway signals support potential cross-population biological relevance External cross-ancestry validation remains needed.	[43]
NSCLC immunotherapy	NSCLC patients receiving immune checkpoint inhibitors Targeted metabolomics + machine learning	Amino acid, glycolytic, and bile acid pathways associated with ICI response Histidine associated with favorable outcomes	Multi-pathway metabolic response signatures identified Cross-ethnic validation not yet reported	[44]
Lung squamous cell carcinoma	79 advanced lung SCC patients Untargeted serum metabolomics LASSO + random forest	Eight-metabolite model predicted chemoimmunotherapy response Validation AUC 0.944	External validation achieved Single-center East Asian cohort without ancestry-stratified analysis	[45]
Breast cancer (ER+), race-stratified	GC-MS plasma metabolomics African American and Non-Hispanic White ER+ breast cancer cohorts Separate Boruta/random forest pipelines	Comparable predictive performance across racial groups (AUC 0.79 vs. 0.78) Distinct race-specific metabolic feature selection observed	Comparable prediction maintained across racial groups despite distinct metabolic signatures Supports population-adaptive metabolomics-based prediction Larger prospective validation remains needed	[46]
Ovarian cancer, multi-site	431 ovarian cancer patients + 133 controls Four North American geographic sites Ensemble ML classifiers	Consistently high predictive performance across all sites (PPV ≥ 93%, NPV ≥ 87%).	Cross-site reproducibility maintained without site-specific recalibration Geographic stability supports potential clinical portability	[47]
dMMR colorectal cancer	Serum metabolomics + machine learning Multi-center neoadjuvant immunotherapy cohort	Five-metabolite predictive model achieved external validation AUC 0.88 SHAP analysis improved interpretability	Multi-center validation supports cross-site reproducibility Ancestry-stratified performance not reported.	[48]
HCC treatment	Baseline plasma metabolomics/lipidomics HCC patients receiving TKI + ICI therapy	Sphingolipid-related lipid species associated with long-term survival.	Lipidomic signatures supported treatment-response stratification Cross-population validation remains needed	[49]

Abbreviations: KTR, kynurenine-to-tryptophan ratio; XKR, xanthurenic acid-to-kynurenine ratio; dMMR, mismatch repair-deficient; ICI, immune checkpoint inhibitor; SCC, squamous cell carcinoma; HCC, hepatocellular carcinoma; TKI, tyrosine kinase inhibitor.

**Table 4:** Selected 2022–2026 metabolic disease studies demonstrating cross-population metabolomics-inclusive AI for equitable precision medicine.

Indication	Cohort/Design	Key Findings	Cross-Population Relevance	Ref.
T2D metabolomic variance decomposition	MESA multiethnic cohort (*n* = 3360) + WHI replication (*n* = 1333) Plasma metabolomics + proteomics Variance decomposition and mediation analysis	Lifestyle and biological factors explained major metabolomic variance Environmentally influenced lipid metabolites associated with T2D risk Lipid/protein mediators accounted for 10–25% of observed racial/ethnic T2D disparities	Multiethnic cohort design with independent replication Findings suggest a contribution of modifiable metabolomic pathways to T2D risk across populations.	[50]
Hispanic/Latino ancestry-specific metabolomics	HCHS/SOL Hispanic/Latino cohort Admixture mapping of 640 circulating metabolites Independent replication cohort	78 ancestry-enriched metabolite associations identified Novel metabolite-locus associations observed across Native American and African ancestry regions	Admixed population design highlighted ancestry-associated metabolomic variation Supports population-aware metabolomics modeling in heterogeneous populations	[51]
T2D metabolomics biomarker discovery, African Americans	Jackson Heart Study African American cohort Targeted + untargeted LC-MS metabolomics External validation in multiethnic MESA	307 metabolites associated with incident T2D 144 associations replicated in MESA Metabolite integration improved prediction performance	Discovery in an underrepresented population with external multiethnic replication Cross-cohort metabolite stability supports broader applicability	[52]
Diabetic microvascular complications, multiethnic Asian to European validation	Multiethnic Southeast Asian SEED cohort External validation in UK Biobank ML prediction of DKD and DR	ML models improved DKD and DR detection compared with traditional logistic regression Improved performance was maintained in external UK Biobank validation	External validation from a multiethnic Southeast Asian cohort to the predominantly European UK Biobank cohort Supports cross-population transferability of metabolomics-informed prediction	[53]
Ancestry-specific T2D effector metabolite mapping	2338 EUR + 417 AFR ancestry participants Metabolome-QTL and proteome-QTL integration with T2D GWAS	Distinct ancestry-associated metabolite effectors identified Shared downstream signaling pathways observed despite metabolite differences	Findings support population-aware metabolomics modeling while suggesting partial mechanistic convergence across ancestries	[54]
Incident T2D prediction	MESA discovery cohort + Rotterdam Study replication cohort Untargeted serum metabolomics	Serum metabolomic profiling improved incident T2D prediction across cohorts	Biomarker stability observed across geographically distinct cohorts.	[55]
Metformin response	Qatar Biobank T2D cohort Pharmacometabolomics of metformin response	Distinct sphingomyelin, glucose metabolism, and microbiome-related signatures identified between responders and non-responders	Population-specific pharmacometabolomic signatures identified Multiethnic replication remains needed	[56]
Sulfonylurea response	Qatar Biobank T2D cohort Pharmacometabolomics of sulfonylurea response	Distinct metabolomic signatures differentiated treatment responders from non-responders	Population-specific response signatures identified External multiethnic validation remains needed	[57]
GLP-1RA/liraglutide response	Liraglutide-treated T2D patients and diet-induced obese mice Plasma metabolomics + machine learning	ML models identified metabolomic patterns associated with liraglutide response	Korean T2D cohort with cross-species validation Cross-ancestry validation in non-Asian populations remains needed	[58]

Abbreviations: DKD, diabetic kidney disease; DR, diabetic retinopathy; GLP-1RA, glucagon-like peptide-1 receptor agonist.

**Table 5:** Selected 2022–2026 studies supporting metabolomics-inclusive AI for transferable prediction in infectious and immune-mediated diseases.

Indication	Cohort/Design	Key Findings	Cross-Population Relevance	Ref.
COVID-19 severity metabolomics meta-analysis	Systematic review/meta-analysis of 22 COVID-19 metabolomics cohorts 2421 participants Untargeted and targeted metabolomics	Amino acid, bile acid, TCA cycle, and taurine-hypotaurine pathways consistently altered with disease severity	Shared metabolic disruption patterns identified across geographically diverse cohorts Supports cross-cohort reproducibility of host-response metabolic signatures	[59]
COVID-19 cross-wave robust metabolites	164 hospitalized COVID-19 patients across two pandemic waves Targeted metabolomics + machine learning	Six-metabolite classifier maintained predictive performance across pandemic waves	Temporal robustness observed despite changes in viral variants and treatment practices External multi-center validation remains needed	[61]
Pulmonary TB diagnosis and treatment monitoring	Ethiopia discovery cohort with external validation in South Africa and Georgia Plasma high-resolution metabolomics Longitudinal treatment monitoring	Kynurenine/tryptophan + retinol signature achieved AUC 0.93–0.97 across cohorts Signature scores decreased during treatment	Performance maintained across geographically distinct cohorts and HIV status groups Supports external reproducibility of host-response metabolomic signatures	[62]
TB multi-cohort lipid biomarkers	Two discovery cohorts + one validation cohort Integrative metabolomics/lipidomics + machine learning	Multi-omics biosignatures achieved external validation AUC 0.77–1.00 Lipid PC(14:0_22:6) identified as a major cross-cohort predictor	External validation across multiple TB differential-diagnosis settings Supports cross-cohort reproducibility of lipid-based TB metabolomic signatures	[63]
Melioidosis diagnosis and prognosis, rural Southeast Asia	Rural northeastern Thailand cohort Untargeted plasma metabolomics Independent validation cohort	12-metabolite diagnostic classifier achieved validation AUC 0.87 Kynurenine pathway activation strongly associated with disease severity	Prospective validation in a resource-limited tropical setting Supports feasibility of metabolomics-based risk stratification in high-burden regions	[64]
Sepsis survival metabolomics + ensemble ML	60 ICU sepsis patients Plasma GC/LC metabolomics Ensemble machine learning ranking	Kynurenine, bile acids, and phenylalanine associated with 28-day mortality Ensemble ML improved metabolite feature prioritization	Mortality-associated kynurenine and bile acid signals overlapped with findings from COVID-19 and TB metabolomics studies External validation remains needed	[65]
Rheumatoid arthritis multi-center metabolomics diagnosis	2863 plasma samples across five independent validation cohorts Targeted metabolomics + five ML algorithms	Six-metabolite classifier achieved robust external validation performance across RA subgroups Diagnostic performance maintained in seronegative RA	Cross-site reproducibility maintained across multiple centers and analytical platforms Supports broader applicability of metabolomics-based immune disease classification	[66]
Community-acquired pneumonia mortality, rural Thailand	107 CAP and 152 non-CAP infection patients Rural northeastern Thailand cohort Untargeted plasma metabolomics + LASSO modeling	Polyamine activation and lipid pathway suppression associated with CAP Four-metabolite mortality signature achieved AUC 0.79	Metabolomics-based mortality prediction demonstrated feasibility within a rural tropical cohort	[67]

Abbreviations: CAP, community-acquired pneumonia.

**Table 6:** Selected 2022–2026 studies supporting metabolomics-inclusive AI across broader precision medicine applications.

Indication	Cohort/Design	Key Findings	Cross-Population Relevance	Ref.
Cardiovascular/CHD risk prediction	900 CHD cases/900 controls Black American, White American, and Chinese cohorts Untargeted plasma metabolomics	24-metabolite risk score associated with incident CHD Improved discrimination beyond conventional cardiovascular risk factors	Comparable metabolite risk associations observed across demographic and lifestyle strata Supports cross-population reproducibility of metabolomics-based cardiovascular risk prediction	[68]
CKD/Nephrology	T2D patients Plasma metabolomics + interpretable machine learning Internal and external validation cohorts	Citrulline and acylcarnitines associated with reduced GFR Metabolomics-based ML improved renal dysfunction prediction	External validation supported reproducibility across cohorts Metabolomics-based renal assessment may reduce reliance on demographic correction factors in eGFR estimation.	[69]
Psychiatry/Cross-ancestry pharmacogenomics of clozapine	UK CLOZUK cohort Five biogeographical ancestry groups Longitudinal pharmacogenomic analysis	Significant ancestry-associated differences in clozapine metabolism observed Polygenic scores explained limited pharmacokinetic variance	Demonstrates substantial cross-ancestry variability in drug metabolism Supports complementary metabolomic CYP phenotyping approaches	[70]
Psychiatry/SSRI response prediction (microbiome-metabolomics)	126 MDD patients receiving SSRI treatment Gut microbiome + metabolomics profiling Machine learning classification	Distinct microbiome-metabolic signatures differentiated treatment responders from non-responders High predictive performance observed in internal testing cohorts	Gut microbiome and metabolomic profiles reflect environmental and host-response variability beyond genomic factors External cross-population validation remains needed	[71]
Maternal-fetal medicine/Early preeclampsia detection	Urine metabolomics + XGBoost Prediction prior to confirmed preeclampsia diagnosis	Four-metabolite urinary signature enabled non-invasive preeclampsia prediction	Non-invasive urine metabolomics may support scalable maternal risk stratification External validation across diverse maternal populations remains needed	[72]
Maternal-fetal medicine/Preeclampsia prediction	Prospective suspected preeclampsia cohort Serum metabolomics + LASSO biomarker selection	Seven-metabolite panel predicted preeclampsia development across discovery and validation cohorts	Prospective two-cohort validation supports reproducibility within independent cohorts External validation in non-Asian populations remains needed	[73]
Cardiovascular/Multi-omics risk prediction	UK Biobank multi-omics AI integrating genomics, proteomics, and metabolomics Six cardiovascular disease endpoints	CardiOmicScore predicted multiple cardiovascular outcomes up to 15 years before onset Metabolomic and proteomic layers improved prediction beyond genomics alone	Predominantly European ancestry cohort Cross-ancestry validation remains needed despite scalable multi-omics framework	[74]
CKD/Nephrology	UK Biobank and additional validation cohorts Blood metabolomic biomarkers Longitudinal CKD follow-up	Metabolite biomarkers improved prediction of incident severe CKD and CKD-related mortality	Multi-cohort validation supported reproducibility across populations Additional non-European validation remains warranted	[75]
Psychiatry/Genomic-metabolomic antidepressant response	MDD antidepressant cohort Plasma metabolomics + germline genomics Network science integration	CLOCK and ARNTL loci associated with metabolomic response signatures Integrated analysis identified biologically distinct antidepressant-response subgroups	Metabolomic-genomic integration identified biologically distinct subgroups beyond symptom-based classification May complement antidepressant response prediction across heterogeneous populations	[76]

Abbreviations: DKD, diabetic kidney disease; DR, diabetic retinopathy.

## Data Availability

No new data were created in this study. All data discussed in this review are available in the cited published literature.

## Abbreviations

The following abbreviations are used in this manuscript:

ADME	Absorption, distribution, metabolism, and excretion
AI	Artificial intelligence
AUC	Area under the receiver operating characteristic curve
CCLE	Cancer Cell Line Encyclopedia
CDR	Cancer drug response
GDSC	Genomics of Drug Sensitivity in Cancer
GNN	Graph neural network
GWAS	Genome-wide association study
IDO1	Indoleamine 2,3-dioxygenase 1
LD	Linkage disequilibrium
LMICs	Low- and middle-income countries
ML	Machine learning
NSCLC	Non-small cell lung cancer
PGx	Pharmacogenomics
PRS	Polygenic risk score
SHAP	SHapley Additive exPlanations
SNP	Single nucleotide polymorphism
TME	Tumor microenvironment

## References

[1] Chiu Y.-C., Chen H.-I.H., Gorthi A., Mostavi M., Zheng S., Huang Y., Chen Y. (2020). Deep learning of pharmacogenomics resources: Moving towards precision oncology. Brief. Bioinform..

[2] Garnett M.J., Edelman E.J., Heidorn S.J., Greenman C.D., Dastur A., Lau K.W., Greninger P., Thompson I.R., Luo X., Soares J. (2012). Systematic identification of genomic markers of drug sensitivity in cancer cells. Nature.

[3] Barretina J., Caponigro G., Stransky N., Venkatesan K., Margolin A.A., Kim S., Wilson C.J., Lehár J., Kryukov G.V., Sonkin D. (2012). The Cancer Cell Line Encyclopedia enables predictive modelling of anticancer drug sensitivity. Nature.

[4] Bycroft C., Freeman C., Petkova D., Band G., Elliott L.T., Sharp K., Motyer A., Vukcevic D., Delaneau O., O’Connell J. (2018). The UK Biobank resource with deep phenotyping and genomic data. Nature.

[5] Haddad A., Radhakrishnan A., McGee S., Smith J.D., Karnes J.H., Venner E., Wheeler M.M., Patterson K., Walker K., Kalra D. (2024). Frequency of pharmacogenomic variation and medication exposures among All of Us Participants. medRxiv.

[6] Dalla-Torre H., Gonzalez L., Mendoza-Revilla J., Lopez Carranza N., Henryk Grzywaczewski A., Oteri F., Dallago C., Trop E., de Almeida B.P., Sirelkhatim H. (2024). The Nucleotide Transformer: Building and Evaluating Robust Foundation Models for Human Genomics. bioRxiv.

[7] Mondello A., Dal Bo M., Toffoli G., Polano M. (2024). Machine learning in onco-pharmacogenomics: A path to precision medicine with many challenges. Front. Pharmacol..

[8] GWAS Diversity Monitor. GWAS Diversity Monitor. https://gwasdiversitymonitor.com/.

[9] Pomales-Matos D.A., Lyerly M., Rivera-Madera A., Echevarría-Bonilla O.L., Álvarez-Cortés M., Henriquez-Quiñones S.E., Reyes-Sosa G.M., Villanueva-Nogueras R.A., Peña-Martínez E.G. (2026). Ancestry gaps in cardiovascular GWAS: A multi-database review of African representation in genomic studies. Front. Genet..

[10] Smith L.A., Cahill J.A., Lee J.-H., Graim K. (2025). Equitable machine learning counteracts ancestral bias in precision medicine. Nat. Commun..

[11] Wu Y., Xie L. (2025). AI-driven multi-omics integration for multi-scale predictive modeling of genotype-environment-phenotype relationships. Comput. Struct. Biotechnol. J..

[12] Stojanović Marković A., Zajc Petranović M., Škarić-Jurić T., Celinšćak Ž., Šetinc M., Tomas Ž., Peričić Salihović M. (2022). Relevance of CYP2D6 Gene Variants in Population Genetic Differentiation. Pharmaceutics.

[13] Koopmans A.B., Braakman M.H., Vinkers D.J., Hoek H.W., van Harten P.N. (2021). Meta-analysis of probability estimates of worldwide variation of CYP2D6 and CYP2C19. Transl. Psychiatry.

[14] Kaye J.B., Schultz L.E., Steiner H.E., Kittles R.A., Cavallari L.H., Karnes J.H. (2017). Warfarin Pharmacogenomics in Diverse Populations. Pharmacother. J. Hum. Pharmacol. Drug Ther..

[15] Shendre A., Dillon C., Limdi N.A. (2018). Pharmacogenetics of warfarin dosing in patients of African and European ancestry. Pharmacogenomics.

[16] Martin A.R., Gignoux C.R., Walters R.K., Wojcik G.L., Neale B.M., Gravel S., Daly M.J., Bustamante C.D., Kenny E.E. (2017). Human Demographic History Impacts Genetic Risk Prediction across Diverse Populations. Am. J. Hum. Genet..

[17] McInnes G., Lavertu A., Sangkuhl K., Klein T.E., Whirl-Carrillo M., Altman R.B. (2020). Pharmacogenetics at Scale: An Analysis of the UK Biobank. Clin. Pharmacol. Ther..

[18] Zanger U.M., Schwab M. (2013). Cytochrome P450 enzymes in drug metabolism: Regulation of gene expression, enzyme activities, and impact of genetic variation. Pharmacol. Ther..

[19] Sharifi-Noghabi H., Zolotareva O., Collins C.C., Ester M. (2019). MOLI: Multi-omics late integration with deep neural networks for drug response prediction. Bioinformatics.

[20] Liu X., Song C., Huang F., Fu H., Xiao W., Zhang W. (2022). GraphCDR: A graph neural network method with contrastive learning for cancer drug response prediction. Brief. Bioinform..

[21] Liu Q., Hu Z., Jiang R., Zhou M. (2020). DeepCDR: A hybrid graph convolutional network for predicting cancer drug response. Bioinformatics.

[22] Kant S., Deepika, Roy S. (2025). Integrative Multi-Omics and Artificial Intelligence: A New Paradigm for Systems Biology. Omics.

[23] Kaddurah-Daouk R., Weinshilboum R.M., Pharmacometabolomics Research Network (2013). Pharmacometabolomics: Implications for Clinical Pharmacology and Systems Pharmacology. Clin. Pharmacol. Ther..

[24] Rashid M.M., Selvarajoo K. (2024). Advancing drug-response prediction using multi-modal and -omics machine learning integration (MOMLIN): A case study on breast cancer clinical data. Brief. Bioinform..

[25] Liu X., Tao Y., Cai Z., Bao P., Ma H., Li K., Li M., Zhu Y., Lu Z.J. (2024). Pathformer: A biological pathway informed transformer for disease diagnosis and prognosis using multi-omics data. Bioinformatics.

[26] Feng R., Xie Y., Lai M., Chen D.Z., Cao J., Wu J. AGMI: Attention-Guided Multi-omics Integration for Drug Response Prediction with Graph Neural Networks. Proceedings of the 2021 IEEE International Conference on Bioinformatics and Biomedicine (BIBM).

[27] Wang C., Zhang M., Zhao J., Li B., Xiao X., Zhang Y. (2023). The prediction of drug sensitivity by multi-omics fusion reveals the heterogeneity of drug response in pan-cancer. Comput. Biol. Med..

[28] Wang C., Lye X., Kaalia R., Kumar P., Rajapakse J.C. (2022). Deep learning and multi-omics approach to predict drug responses in cancer. BMC Bioinform..

[29] Dankwa-Mullan I., Weeraratne D. (2022). Artificial Intelligence and Machine Learning Technologies in Cancer Care: Addressing Disparities, Bias, and Data Diversity. Cancer Discov..

[30] Drozda K., Wong S., Patel S.R., Bress A.P., Nutescu E.A., Kittles R.A., Cavallari L.H. (2015). Poor warfarin dose prediction with pharmacogenetic algorithms that exclude genotypes important for African Americans. Pharmacogenet. Genom..

[31] Asiimwe I.G., Pirmohamed M. (2022). Ethnic Diversity and Warfarin Pharmacogenomics. Front. Pharmacol..

[32] Seyyed-Kalantari L., Zhang H., McDermott M.B.A., Chen I.Y., Ghassemi M. (2021). Underdiagnosis bias of artificial intelligence algorithms applied to chest radiographs in under-served patient populations. Nat. Med..

[33] Waqas A., Tripathi A., Ahmed S., Mukund A., Farooq H., Johnson J.O., Stewart P.A., Naeini M., Schabath M.B., Rasool G. (2025). Self-Normalizing Multi-Omics Neural Network for Pan-Cancer Prognostication. Int. J. Mol. Sci..

[34] Scott M., Lundberg S.-I.L. A Unified Approach to Interpreting Model Predictions. Proceedings of the Advances in Neural Information Processing Systems.

[35] Rudin C. (2019). Stop explaining black box machine learning models for high stakes decisions and use interpretable models instead. Nat. Mach. Intell..

[36] Acosta J.N., Falcone G.J., Rajpurkar P., Topol E.J. (2022). Multimodal biomedical AI. Nat. Med..

[37] Li B.S., Cai T., Duan R. (2023). Targeting Underrepresented Populations in Precision Medicine: A Federated Transfer Learning Approach. Ann. Appl. Stat..

[38] Casaletto J., Bernier A., McDougall R., Cline M.S. (2023). Federated Analysis for Privacy-Preserving Data Sharing: A Technical and Legal Primer. Annu. Rev. Genom. Hum. Genet..

[39] Zack M., Stupichev D.N., Moore A.J., Slobodchikov I.D., Sokolov D.G., Trifonov I.F., Gobbs A. (2025). Artificial Intelligence and Multi-Omics in Pharmacogenomics: A New Era of Precision Medicine. Mayo Clin. Proc. Digit. Health.

[40] Kairouz P., McMahan H.B., Avent B., Bellet A., Bennis M., Bhagoji A.N., Bonawitz K., Charles Z., Cormode G., Cummings R. (2021). Advances and Open Problems in Federated Learning. Found. Trends Mach. Learn..

[41] Liu K., Altman R.B. (2025). Conditional Generative Models for Synthetic Tabular Data: Applications for Precision Medicine and Diverse Representations. Annu. Rev. Biomed. Data Sci..

[42] Wyllie S., Shumailov I., Papernot N. Fairness Feedback Loops: Training on Synthetic Data Amplifies Bias. Proceedings of the 2024 ACM Conference on Fairness, Accountability, and Transparency.

[43] Sun R., Fei F., Wang M., Jiang J., Yang G., Yang N., Jin D., Xu Z., Cao B., Li J. (2023). Integration of metabolomics and machine learning revealed tryptophan metabolites are sensitive biomarkers of pemetrexed efficacy in non-small cell lung cancer. Cancer Med..

[44] Lee S.H., Kim S., Lee J., Kim Y., Joo Y., Heo J.Y., Lee H., Lee C., Hwang G.S., Park H. (2024). Comprehensive metabolomic analysis identifies key biomarkers and modulators of immunotherapy response in NSCLC patients. Drug Resist. Updat..

[45] Zheng L., Nie W., Wang S., Yang L., Hu F., Ma M., Cheng L., Lu J., Zhang B., Xu J. (2025). Metabolomic machine learning-based model predicts efficacy of chemoimmunotherapy for advanced lung squamous cell carcinoma. Front. Immunol..

[46] Santaliz-Casiano A., Mehta D., Danciu O.C., Patel H., Banks L., Zaidi A., Buckley J., Rauscher G.H., Schulte L., Weller L.R. (2023). Identification of metabolic pathways contributing to ER+ breast cancer disparities using a machine-learning pipeline. Sci. Rep..

[47] Ban D., Housley S.N., Matyunina L.V., McDonald L.D., Bae-Jump V.L., Benigno B.B., Skolnick J., McDonald J.F. (2024). A personalized probabilistic approach to ovarian cancer diagnostics. Gynecol. Oncol..

[48] Ma T., Zhang W., Pan Y., Long G., Mi X., Jiang J., Bai F., Zhang H., Hu T., Zeng Z. (2026). A serum metabolite-based machine learning model predicts response to neoadjuvant immunotherapy in mismatch repair-deficient colorectal cancer. Front. Oncol..

[49] Guan S., Yuan G., Xian T., Chen Y., Li R., Zhang G., Chan S., Fang J.-H., Huang M., Bi H. (2025). Metabolomics and lipidomics predictor of survival in hepatocellular carcinoma patients receiving tyrosine kinase inhibitor and immune checkpoint inhibitor combination therapy. Drug Metab. Dispos..

[50] Sevilla-González M., Wang N., Hanson P.A., Bebo A., Hitchcock D., Hsu S., Westerman K.E., Cromer S.J., Barry V.G., Borns-Weil Y. (2025). Dissecting Genetic and Environmental Determinants of Plasma Molecular Signatures and Their Link to Type 2 Diabetes Risk. medrxiv.

[51] Reynolds K.M., Horimoto A.R.V.R., Lin B.M., Zhang Y., Kurniansyah N., Yu B., Boerwinkle E., Qi Q., Kaplan R., Daviglus M. (2023). Ancestry-driven metabolite variation provides insights into disease states in admixed populations. Genome Med..

[52] Chen Z.Z., Pacheco J.A., Gao Y., Deng S., Peterson B., Shi X., Zheng S., Tahir U.A., Katz D.H., Cruz D.E. (2022). Nontargeted and Targeted Metabolomic Profiling Reveals Novel Metabolite Biomarkers of Incident Diabetes in African Americans. Diabetes.

[53] He F., Ling C.N.Y., Nusinovici S., Cheng C.-Y., Wong T.Y., Li J., Sabanayagam C. (2024). Development and External Validation of Machine Learning Models for Diabetic Microvascular Complications: Cross-Sectional Study With Metabolites. J. Med. Internet Res..

[54] Yang C., Gorijala P., Timsina J., Wang L., Liu M., Wang C., Brock W., Wang Y., Urano F., Sung Y.J. (2025). European and African ancestry-specific plasma protein-QTL and metabolite-QTL analyses identify ancestry-specific T2D effector proteins and metabolites. Nat. Commun..

[55] Jiang X., Zhu F., Graça G., Du X., Ran J., Ahmadizar F., Wood A.C., Zhou Y., Scholtens D.M., Farzaneh A. (2025). Serum Metabolomic Profiling of Incident Type 2 Diabetes Mellitus in the Multi-ethnic Study of Atherosclerosis and Rotterdam Study. J. Clin. Endocrinol. Metab..

[56] Naja K., Anwardeen N., Al-Hariri M., Al Thani A.A., Elrayess M.A. (2023). Pharmacometabolomic Approach to Investigate the Response to Metformin in Patients with Type 2 Diabetes: A Cross-Sectional Study. Biomedicines.

[57] Naja K., Anwardeen N., Bashraheel S.S., Elrayess M.A. (2024). Pharmacometabolomics of sulfonylureas in patients with type 2 diabetes: A cross-sectional study. J. Pharm. Pharm. Sci..

[58] Park S., Kim E.K. (2024). Machine Learning-Based Plasma Metabolomics in Liraglutide-Treated Type 2 Diabetes Mellitus Patients and Diet-Induced Obese Mice. Metabolites.

[59] Bi C., He J., Yuan Y., Che S., Cui T., Ning L., Li Y., Dou Z., Han L. (2025). Metabolomic characteristics and related pathways in patients with different severity of COVID-19: A systematic review and meta-analysis. J. Glob. Health.

[60] Richard V.R., Gaither C., Popp R., Chaplygina D., Brzhozovskiy A., Kononikhin A., Mohammed Y., Zahedi R.P., Nikolaev E.N., Borchers C.H. (2022). Early Prediction of COVID-19 Patient Survival by Targeted Plasma Multi-Omics and Machine Learning. Mol. Cell. Proteom..

[61] Lewis H.-M., Liu Y., Frampas C.F., Longman K., Spick M., Stewart A., Sinclair E., Kasar N., Greener D., Whetton A.D. (2022). Metabolomics Markers of COVID-19 Are Dependent on Collection Wave. Metabolites.

[62] Collins J.M., Bobosha K., Narayanan N., Gandhi N.R., Day C.L., Rengarajan J., Kempker R.R., Lau M.S.Y., Nellis M., Tukvadze N. (2025). A Plasma Metabolic Signature to Diagnose Pulmonary Tuberculosis and Monitor Treatment Response. J. Infect. Dis..

[63] Tien N.T.N., Yen N.T.H., Phat N.K., Anh N.K., Thu N.Q., Eunsu C., Kim H.-S., Hoa V.D., Nguyen D.N., Kim D.H. (2025). Multiomics and Machine Learning Identify Immunometabolic Biomarkers for Active Tuberculosis Diagnosis Against Nontuberculous Mycobacteria and Latent Tuberculosis Infection. J. Proteome Res..

[64] Xia L., Hantrakun V., Teparrukkul P., Wongsuvan G., Kaewarpai T., Dulsuk A., Day N.P.J., Lemaitre R.N., Chantratita N., Limmathurotsakul D. (2023). Plasma Metabolomics Reveals Distinct Biological and Diagnostic Signatures for Melioidosis. Am. J. Respir. Crit. Care Med..

[65] Kosyakovsky L.B., Somerset E., Rogers A.J., Sklar M., Mayers J.R., Toma A., Szekely Y., Soussi S., Wang B., Fan C.-P.S. (2022). Machine learning approaches to the human metabolome in sepsis identify metabolic links with survival. Intensive Care Med. Exp..

[66] Tang J., Jiang R., Gao H., Xia J., Ma Y., Han Z., Yu H., Zhang Y., Xie F., Sheng H. (2025). Development and multi-center validation of machine learning models based on targeted metabolomics for rheumatoid arthritis. J. Transl. Med..

[67] Coston T.D., Xia L., Wright S.W., Hantrakun V., Chamnan P., Wongsuvan G., Phunpang R., Dulsuk A., Thiansukhon E., Shojaie A. (2025). Pneumonia-specific plasma metabolite profiles among patients hospitalised with infection in Southeast Asia. ERJ Open Res..

[68] Deng K., Gupta D.K., Shu X.-O., Lipworth L., Zheng W., Cai H., Cai Q., Yu D. (2024). Circulating Metabolite Profiles and Risk of Coronary Heart Disease Among Racially and Geographically Diverse Populations. Circ. Genom. Precis. Med..

[69] An T.-F., Zhang Z.-P., Xue J.-T., Luo W.-M., Li Y., Fang Z.-Z., Zong G.-W. (2024). Interpretable machine learning identifies metabolites associated with glomerular filtration rate in type 2 diabetes patients. Front. Endocrinol..

[70] Pardiñas A.F., Kappel D.B., Roberts M., Tipple F., Shitomi-Jones L.M., King A., Jansen J., Helthuis M., Owen M.J., O’Donovan M.C. (2023). Pharmacokinetics and pharmacogenomics of clozapine in an ancestrally diverse sample: A longitudinal analysis and genome-wide association study using UK clinical monitoring data. Lancet Psychiatry.

[71] Jiang Y., Qu Y., Shi L., Ou M., Du Z., Zhou Z., Zhou H., Zhu H. (2024). The role of gut microbiota and metabolomic pathways in modulating the efficacy of SSRIs for major depressive disorder. Transl. Psychiatry.

[72] Chen Q., Qian Y., Feng M., Zhang H., Xie H. (2025). Integrating urine metabolomic biomarkers and machine learning algorithms to predict preeclampsia. Eur. J. Med. Res..

[73] Cao Y., Meng L., Wang Y., Zhao S., Zheng Y., Ran R., Du J., Wu H., Han J., Xu Z. (2025). Large-scale prospective serum metabolomic profiling reveals candidate predictive biomarkers for suspected preeclampsia patients. Sci. Rep..

[74] Luo Y., Zhang N., Yang J., Cui M., Tsoi K.K.F., Lip G.Y.H., Liu T., Zhang Q. (2026). AI-based multiomics profiling reveals complementary omics contributions to personalized prediction of cardiovascular disease. Nat. Commun..

[75] Nusinovici S., Li H., Chong C., Yu M., Sørensen I.M.H., Bisgaard L.S., Christoffersen C., Bro S., Liu S., Liu J.J. (2024). Blood biomarkers improve the prediction of prevalent and incident severe chronic kidney disease. J. Nephrol..

[76] Grant C.W., Wilton A.R., Kaddurah-Daouk R., Skime M., Biernacka J., Mayes T., Carmody T., Wang L., Lazaridis K., Weinshilboum R. (2022). Network science approach elucidates integrative genomic-metabolomic signature of antidepressant response and lifetime history of attempted suicide in adults with major depressive disorder. Front. Pharmacol..

[77] Fujisaka S., Avila-Pacheco J., Soto M., Kostic A., Dreyfuss J.M., Pan H., Ussar S., Altindis E., Li N., Bry L. (2018). Diet, Genetics, and the Gut Microbiome Drive Dynamic Changes in Plasma Metabolites. Cell Rep..

